# Hsp90 co-chaperones, FKBP52 and Aha1, promote tau pathogenesis in aged wild-type mice

**DOI:** 10.1186/s40478-021-01159-w

**Published:** 2021-04-08

**Authors:** Marangelie Criado-Marrero, Niat T. Gebru, Danielle M. Blazier, Lauren A. Gould, Jeremy D. Baker, David Beaulieu-Abdelahad, Laura J. Blair

**Affiliations:** 1 USF Health Byrd Alzheimer’s Institute, University of South Florida, Tampa, FL 33613 USA; 2 Department of Molecular Medicine, Morsani College of Medicine, University of South Florida, Tampa, FL 33620 USA; 3 Research Service, James A Haley Veterans Hospital, 13000 Bruce B Downs Blvd, Tampa, FL 33612 USA

**Keywords:** Tau, FKBP52, Aha1, Molecular chaperones, Alzheimer’s disease, Neuroinflammation

## Abstract

The microtubule associated protein tau is an intrinsically disordered phosphoprotein that accumulates under pathological conditions leading to formation of neurofibrillary tangles, a hallmark of Alzheimer’s disease (AD). The mechanisms that initiate the accumulation of phospho-tau aggregates and filamentous deposits are largely unknown. In the past, our work and others’ have shown that molecular chaperones play a crucial role in maintaining protein homeostasis and that imbalance in their levels or activity can drive tau pathogenesis. We have found two co-chaperones of the 90 kDa heat shock protein (Hsp90), FK506-binding protein 52 (FKBP52) and the activator of Hsp90 ATPase homolog 1 (Aha1), promote tau aggregation in vitro and in the brains of tau transgenic mice. Based on this, we hypothesized that increased levels of these chaperones could promote tau misfolding and accumulation in the brains of aged wild-type mice. We tested this hypothesis by overexpressing Aha1, FKBP52, or mCherry (control) proteins in the hippocampus of 9-month-old wild-type mice. After 7 months of expression, mice were evaluated for cognitive and pathological changes. Our results show that FKBP52 overexpression impaired spatial reversal learning, while Aha1 overexpression impaired associative learning in aged wild-type mice. FKBP52 and Aha1 overexpression promoted phosphorylation of distinct AD-relevant tau species. Furthermore, FKBP52 activated gliosis and promoted neuronal loss leading to a reduction in hippocampal volume. Glial activation and phospho-tau accumulation were also detected in areas adjacent to the hippocampus, including the entorhinal cortex, suggesting that after initiation these pathologies can propagate through other brain regions. Overall, our findings suggest a role for chaperone imbalance in the initiation of tau accumulation in the aging brain.

**Supplementary Information:**

The online version contains supplementary material available at 10.1186/s40478-021-01159-w.

## Introduction

Alzheimer’s disease (AD) is a progressive neurodegenerative disease and the most common cause of dementia in older adults [45]. The accumulation of tau, encoded by the *MAPT* gene, in the brains of AD patients is one of the pathological drivers of disease progression [86]. Tau is an abundant protein in the peripheral and central nervous systems that functions to stabilize neuronal microtubules [54, 65]. Under pathological conditions, tau disassociates from the microtubules, misfolds, becomes hyperphosphorylated, and forms aggregates. These conformational changes in tau are thought to contribute to disease progression and neurodegeneration in AD and other tauopathies [51, 91, 93].

As an effort to better understand tau-mediated pathological and neurobehavioral outcomes in AD, many transgenic mouse lines have been created that show tau accumulation, some with cognitive deficits and neuronal loss, which recapitulate aspects of the tauopathic brain [73, 82, 104]. However, they also have limitations often including the need to use tau mutations and/or overexpression [82, 104], which are not found in the AD brain. Drift in the reliability of the line over time and between groups, and genetic disruptions that contribute to the neurodegenerative features have also been reported [35, 49, 52]. The contribution of aging, the strongest risk factor for tauopathies including AD [1], is not appropriately modeled in these mice. Mouse models have also been created that express human tau at endogenous levels [3, 43, 44, 81], which are useful for studying age-related aspects of tauopathy. While these models have some tau accumulation, they still do not capture the cascade of pathological events in the AD brain. Because of this many groups are now potentiating the pathology in these models by injecting pathological tau seeds [21, 71], which is informative at understanding the propagation of tau through the brain but does not allow us to understand which events initiate tau accumulation under normal physiological conditions.

As we age, our cells become less efficient at maintaining proper protein folding and disposal [56]. Cells employ highly conserved proteins known as molecular chaperones to maintain protein homeostasis [42, 55]. Molecular chaperones regulate protein synthesis, folding, trafficking, and assembly of multiprotein complexes to maintain a balanced protein quality control system [12]. They also facilitate degradation of terminally modified or misfolded proteins**,** an essential role within the proteostasis network [68, 91]. Major imbalances in the chaperone system have been reported in the aged brain, where 32% of chaperones are downregulated and almost 20% of chaperones are upregulated [14]. Those that decrease are predominantly ATP-dependent chaperones required for protein folding whereas those that increase are more commonly ATP-independent chaperones and co-chaperones [14]. Further changes in molecular chaperone expression have also been shown in the AD brain [15, 33, 58]. These age- and AD-associated changes in molecular chaperones may be early events that contribute to the cascade of pathologies associated with AD. In fact, work from our group and others has shown that high expression of certain molecular chaperones can drive the aggregation of tau [11, 17, 23, 36, 53, 90]. A well-studied and highly conserved chaperone is the 90 kDa heat shock protein (Hsp90) that maintains cellular homeostasis [63, 80]. However, under pathological conditions, Hsp90 can promote the accumulation of tau [11, 23, 101]. Specific actions on tau protein will depend on the association of Hsp90 with many other chaperones forming a heterocomplex [11, 18, 19, 74, 90]. In this study, we investigated the effects of two Hsp90 co-chaperones on tau in the aged brain: the activator of 90 kDa heat shock protein ATPase homolog 1 (Aha1) and the 52 kDa FK506-binding protein (FKBP52). Recently, we have found that these proteins cause increased tau accumulation in the brains of tau transgenic mice [19, 90].

Aha1 is a cytosolic Hsp90 co-chaperone that promotes Hsp90 ATPase activity [62]. In the past, we have shown that the overexpression of Aha1 in the hippocampus of tau transgenic mice increased oligomeric and insoluble tau, which was complemented by neuronal loss and cognitive impairments [91]. There was also a modest increase, although not significant, in soluble tau levels in the hippocampus of 5-month-old wild-type mice after 3 months of Aha1 overexpression. In postmortem AD brains, Aha1 colocalized with pathogenic tau and correlated with disease progression [90]. In addition to tau, Aha1 stabilizes other Hsp90 clients including mutant cystic fibrosis transmembrane conductance regulator, CFTR, which causes cystic fibrosis [59, 100], and mutant melanocortin-4 receptor, another misfolded transmembrane protein [67]. Aha1 may also have a role in RNA processing and DNA repair [96].

FKBP52 is an Hsp90 co-chaperone highly expressed in neurons and a regulator of steroid hormone complexes [76, 103]. Roles for FKBP52 in regulation of nuclear trafficking of multiprotein complexes—progesterone, glucocorticoid, and androgen receptors have been described [92]. FKBP52 can regulate microtubule assembly by directly binding to tau, preferentially in its hyperphosphorylated form [18]. FKBP52 promotes the aggregation of various tau species, including wild-type and P301L recombinant tau, leading to the formation of tau oligomers and fibrils [18, 37]. The effect of FKBP52, but not Aha1, on tau aggregation is independent of Hsp90 and ATP [18, 19, 36, 71]. In a recent study, we found that FKBP52 overexpression in the hippocampus of the rTg4510 tau transgenic mouse promotes spatial memory impairments and neuronal loss [19]. Different from Aha1, FKBP52 was found to be colocalized with normal but not pathological tau in the frontal cortex of human AD brains [38]. Interestingly, the same study revealed that FKBP52 levels were lower in AD brains when compared to control subjects. However, the cause and consequences of this decrease in FKBP52 levels are still not clearly understood. Aha1 and FKBP52 were associated with reduced cognitive trajectory, in a recent study investigating the association of proteins with the stability of cognition through aging [102]. Together, this led us to hypothesize that imbalance in these chaperones may occur at different timings before or during disease progression and that these chaperones may be useful to model if chaperone dysregulation is sufficient to trigger tau misfolding in the aging brain.

Aha1 and FKBP52 can bind to Hsp90 simultaneously, but they compete with other Hsp90 co-chaperones [96]. The composition of Hsp90 heterocomplexes affects client maturation and activity [10]. In particular, the activity of steroid hormone complexes, like the glucocorticoid (GR) and mineralocorticoid (MR) receptors, are directly modulated by the binding of these co-chaperones to Hsp90. Aha1 competes with GR to bind Hsp90 [66]. Hsp90 inhibition reduces GR and MR activity [6], but HAM-1, an inhibitor of Hsp90/Aha1 client processing, did not alter GR activity, while MR activity was reduced [95]. This highlights that Aha1 is important for the maturation of MR, but not GR. In contrast, FKBP52 has been shown to increase GR, but not MR, activity [27, 34]. However, FKBP52 is important for the nuclear localization of MR, which precedes its activation [34]. Given their overlapping effects on tau accumulation and differing effects steroid hormone complexes, Aha1 and FKBP52 are prime candidates to investigate how chaperone imbalance may contribute to declined proteostasis during aging.

The present study evaluated whether upregulation of Aha1 or FKBP52 could initiate a pathological cascade to cause tau accumulation in the brains of aged wild-type mice. In addition to tau, we examined the effects on neuroinflammation and neurodegeneration in the hippocampus and its adjacent cortical areas. Behavioral tasks of learning and memory were also performed to test cognitive impairments. We found that high levels of these chaperones were enough to promote pathological and phenotypic consequences. Our findings are highly relevant to understand how protein dysregulation during normal aging may promote the molecular and behavioral phenotype seen in tauopathies.

## Materials and methods

### Virus production

Aha1, FKBP52, and mCherry were subcloned into pTR12.1-MCS vector containing a short hybrid CMV/chicken β-actin promoter [20]. All plasmids were confirmed by sequencing. Aha1-pTR12.1, FKBP52-pTR12.1 or mCherry-pTR12.1 were co-transfected with helper plasmids pFΔ6 and pAAV9 into HEK293T using polyethyleneimine to generate adeno-associated virus serotype 9 (AAV9) particles. After 72 h, the recombinant virus was harvested by three cycles of freeze–thaw between dry-ice and 37 °C water bath. The crude lysate was clarified by centrifugation at 4000×*g* for 20 min and purified using four iodixanol gradients. After concentration to 200 µL, a SYBR-green-based real time PCR was used to determine the viral titer in genomes/mL.

### Mouse colony and viral injections

Wild-type mice (129S6 X FVB/N background) were obtained from our colony at the University of South Florida vivarium. They were housed up to five per cage, maintained under standard conditions with a 12-h light/dark cycle, and had free access to food and water. Animal experiments were carried out accordingly with the NIH Guide for the Care and Use of Laboratory Animals and approved by the University of South Florida Institutional Animal Care and Use Committee (IACUC). At 9 months of age, mice (N = 6 for each condition) were injected with AAV9 expressing Aha1, FKBP52, or mCherry (control) using a robotic stereotaxic surgery unit (Neurostar GmbH, Tubingen, Germany). A total of 2 µL of 1 × 10^12^ genomes/mL viral particles was delivered into each injection site using a high precision syringe (Hamilton 801 RN, HT7642-01, College Park, Georgia) with convection enhanced delivery [16] at the following coordinates: hippocampi (X = ± 3.6, Y = − 3.5, and Z = + 2.68) and cortex (X = ± 2.2, Y = + 1.7, and Z = + 3.0). After 7 months of expression, brain tissues were harvested from 16-month-old mice following transcardial perfusion using 0.9% saline solution.

### Immunohistochemistry

Free-floating tissue was stained as previously described [11, 22]. Briefly, tissue sections were incubated in PBS supplemented with 10% MeOH and 3% H_2_O_2_ to block endogenous peroxidases. Following PBS washes, tissue was permeabilized by 0.2% Triton-X-100 with 1.83% lysine and 4% goat serum in PBS for 30 min. Tissue was incubated overnight at room temperature using the following primary antibodies: Total tau (Dako; 1:100,000, Aligent A002401, Santa Clara, CA, USA), FKBP52 (1:1000, R&D Systems MAB4095, Minneapolis, MN, USA), Aha1 (1:100, Stressmarq Biosciences SMC172D, Victoria, British Columbia, Canada), pT231 Tau (1:300, Anaspec AS-55313, Fremont, CA, USA), AT8-biotin (S202/T205; 1:5000, Thermo Fisher ENMN1020B, Waltham, MA, USA), pS396 Tau (1:5000, Anaspec AS-54977, Fremont, CA, USA), T22 (1:15,000, Millipore ABN454, Burlington, MA, USA), GFAP (1:10,000, Millipore MAB360, Burlington, MA, USA), and IBA1 (1:10,000, WAKO 019-19741, Chuo-Ku, Tokyo, Japan). After washes, sections were incubated for two hours with the corresponding secondary antibodies (Southern Biotech, Birmingham, AL, USA). A Vectastain ABC kit (Vector Laboratories, PK-4000, San Francisco, CA, USA) was used to increase visibility. This was followed by washes, incubation with 0.05% diaminobenzidine plus 0.5% nickel sulfate hexahydrate in TBS and development with 0.03% H_2_O_2_. Following washes, tissue sections were mounted and allowed to dry overnight before dehydration in alcohol gradients. Slides were cleared by Histoclear then coverslipped with DPX mountant. Tissue sections were mounted on glass slides, dried overnight, and coverslipped using Prolong Gold Antifade Reagent (Invitrogen P36934) for mCherry detection.

### Gallyas silver staining

Tissue slices were mounted on glass slides and dried overnight. Sections were washed twice with distilled water and incubated in 5% periodic acid for five minutes. After two five-minute washes with distilled water, sections were incubated in alkaline silver iodide solution (1 M sodium hydroxide, 0.6 M potassium iodide, 0.053% silver nitrate) for one minute. Sections were then washed with 0.5% acetic acid for 10 min. Staining was developed by combining solutions A (5% sodium carbonate), B (0.024 M ammonium nitrate, 0.012 M silver nitrate, 0.003 M tungstosilicic acid), and C (0.024 M ammonium nitrate, 0.012 M silver nitrate, 0.003 M tungstosilicic acid, 0.25% formaldehyde) in a 10:3:7 ratio. Adding B first to A and then adding C. Slides were submerged in this solution with constant mixing for 10 min. The slides were then washed in 0.5% acetic acid for three min, then distilled water for five min. Slides were then incubated in 0.1% gold chloride for 5 min and again rinsed with distilled water. Slides were incubated in 0.1% sodium thiosulphate solution for 5 min and washed with tap water. Finally, they were rapidly dehydrated using EtOH gradients and coverslipped with xylenes.

### Stereology and high magnification images

Tissue was stained with biotinylated NeuN (1:100, EMD Millipore MAB377B) as described above, but without 0.5% nickel sulfate hexahydrate and TBS. Tissues were mounted and before coverslipping they were counterstained with 0.05% cresyl violet (Nissl) to quantify neurons. Only those positive for both NeuN and Nissl stains in the Cornu ammonis 1 (CA1) region of the hippocampus were counted. A stereology workstation consisting of a modified Leica DM4000B light microscope with a Prior motorized stage was used to outline the area using distinct landmarks in the brain at 4× magnification [2, 69]. Neurons in this region were counted using randomly designated areas in the computer-generated grid using a 100 × oil immersion objective. Neurons were counted when they were located within the three-dimensional dissectors or touching the inclusion lines, and the top 1 µm and bottom 1 µm of tissue were excluded. High magnification images of tau staining in the hippocampus were also taken using the high power (100x, NA 1.3) oil immersion objective on this microscope.

### Image analysis and statistics

Zeiss Axio Scan.Z1 (ZEISS Microscopy, Munich, Germany) was used to image all tissue slides. Pixel-based bright-field image analysis software, NearCYTE (http://nearcyte.org), was used to outline a region of interest within the tissue. Threshold was adjusted to select only stain positive cells. Parametric segmentation method was used to create a file for each stain and applied using a batch processing option to enable the quantification of percent area ratio of positive cells within the selected regions of interest for each slide. Mean area ratio was used for each group for graphical representation. Significant outliers were detected using GraphPad Prism 8.0 (GraphPad Software, San Diego, CA, USA).

### Open field

Prior to each behavioral test, mice were acclimated to the testing room for a minimum of 30 min. Then, individual mice were placed in the center of the box and allowed to explore for a period of 10 min while video recorded. Time spent in the center and corners as well the total distance travelled by each mouse was monitored and analyzed using the ANY-maze (Stoelting, www.anymaze.com) video tracking software operated by a blinded observer.

### Radial-arm water maze (RAWM)

The RAWM task was used to test for spatial learning and memory deficits in these mice. A black round swimming pool containing 6 radial metal arms was filled with water. A submerged platform (1 cm below surface) was placed in the goal arm. During the first day, each animal was permitted swim for up to 60 s per trial to locate the platform (length of each trial). A visible and hidden platform was alternated between trials. During the second day, mice were trained leaving a hidden platform throughout the trials. The last day of training, the platform was moved to the counter-goal arm (new goal). Each day consisted in 12 trials (4 block sessions, 3 trials each). A blind observer manually scored the number of errors in each trial. An error was defined as entry into incorrect arm or no arm entry within the first 15 s. If an animal failed to locate the platform in the allow time, they were manually led to the platform. Once mice reached platform, they were allowed to observe the spatial cues for one minute.

### Fear conditioning

Associated learning and memory is mainly regulated by hippocampal projections. Thus, deficits in associative learning and contextual memory were evaluated by training the mice to associate a conditioned stimulus (70-dB tone) presented for 30 s that co-terminated with an unconditioned stimulus (2 s, 0.5 mA foot shock). Twenty-four hours later, mice were placed into the same chamber as the first day and monitored for 3 min without tone or shock exposure (Context). On the next day, mice were placed back into a novel context for three minutes and exposed to a 70-dB tone for three minutes without a shock (Cued). Freezing (lack of movement) was evaluated every 10 s by a blind observer to the experiment. Averages of percent freezing by min are shown.

#### Statistical analysis

Statistical analysis for open field, immunostained tissues, and stereology was performed using one-way analysis of variance (ANOVA). RAWM and fear tests were analyzed by repeated measures two-way ANOVA. When significant, the analysis was followed by Tukey’s *post-hoc* test using GraphPad Prism 8. Data normal distribution was confirmed by Shapiro–Wilk test. Spearman rank correlations were calculated to examine associations between tau-related proteins, neuronal count, and neuroinflammatory cell markers. Significant difference was considered when p values where less than 0.05 (*), 0.01 (**) and 0.001 (***) as described in figure legends. Data is presented as the standard error of the mean (± SEM).

## Results

### High FKBP52 levels impair spatial reversal learning, while overexpression of Aha1 impairs associative learning in aged wild-type mice

**Fig. 1:**
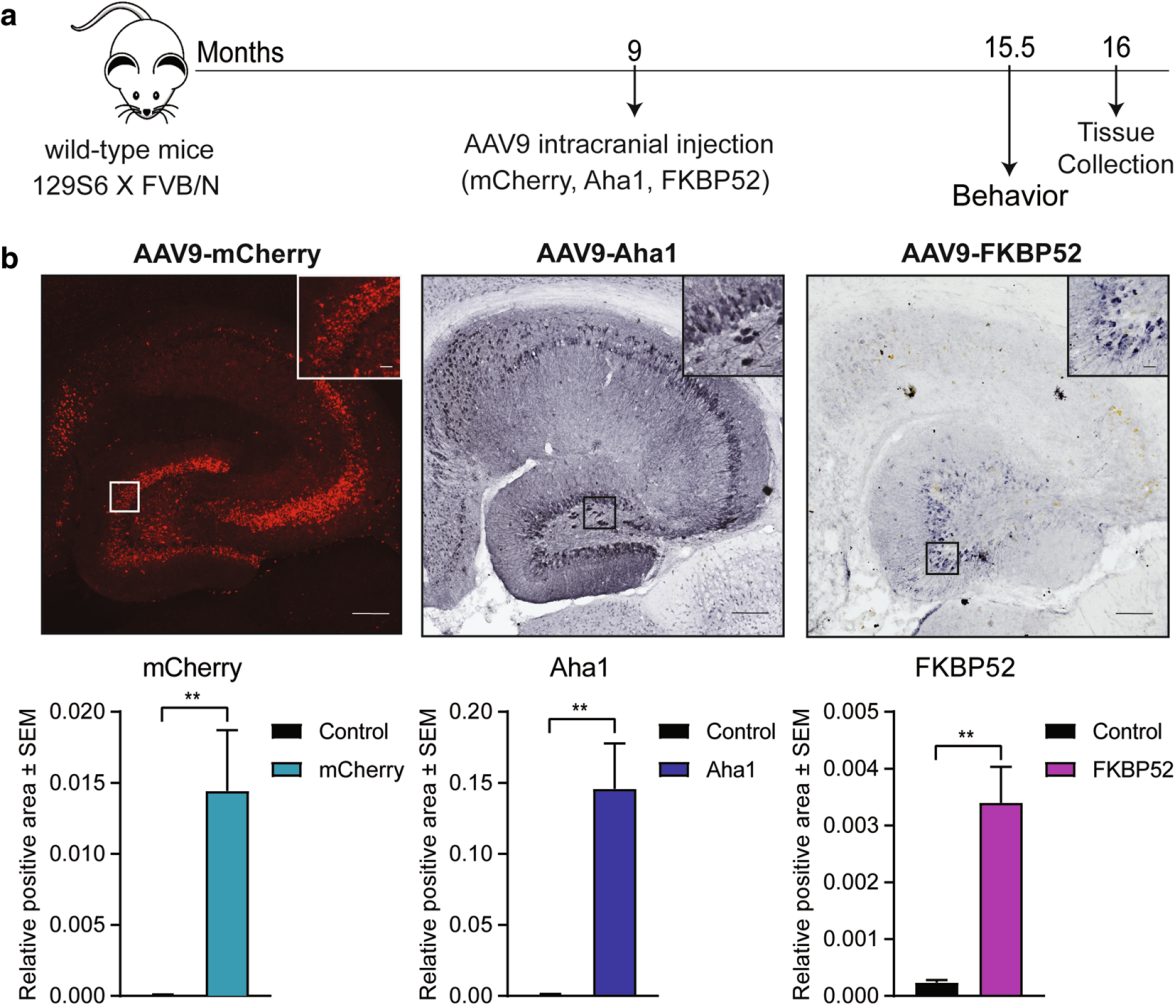
AAV9 injection timeline and expression validation. (**a**) Timeline of bilateral hippocampal injections with AAV9-mCherry (control), AAV9-Aha1, or AAV9-FKBP52. At 15.5 months of age, mice were exposed to cognitive behavioral testing followed by brain collection for immunohistochemistry analysis. N = 6/AAV. (**b**) Representative images and quantification of viral expression after 7 months of injection in hippocampal sections from wild-type mice. Relative intensity was analyzed by Student’s unpaired t-test comparing outside (control) and inside areas of injection. Statistical significance is indicated by **p < 0.01. Results are represented as standard error of the mean (± SEM). Scale bar represents 100 µm; inset scale represents 10 µm. AAV9, adeno-associated virus serotype 9

**Fig. 2:**
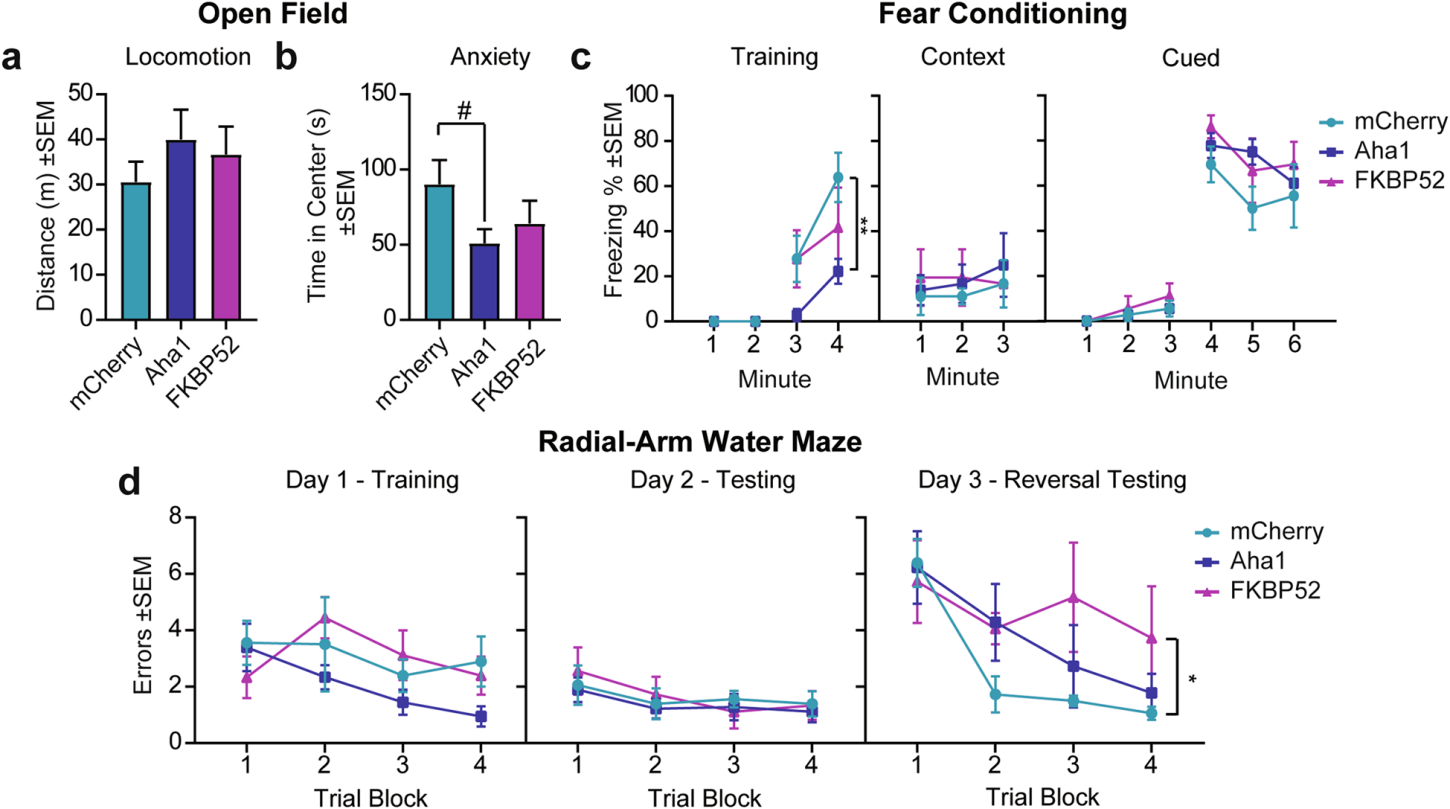
FKBP52 impairs spatial reversal learning, while Aha1 impairs associative learning. AAV9-mCherry, AAV9-Aha1, and AAV9-FKBP52 injected wild-type mice were tested using the open field for (**a**) locomotor activity—indicated by total distanced traveled- and (**b**) anxiety-like behavior—indicated by total time spent in the center. (**c**) Percent time freezing was measured in fear conditioning—a behavioral task of associative learning and memory. (**d**) Spatial learning and memory using the radial-arm water maze (RAWM) test: Day 1-Training, Day 2-Testing, and Day 3-Reversal Testing. N = 6/AAV. Open field was analyzed by a one-way ANOVA while fear conditioning and RAWM by repeated measures two-way ANOVA. This was followed by Tukey post-hoc test when group significance was found. Results represented as standard error of the mean (± SEM). ^#^*p* = 0.07, *p < 0.05, **p < 0.01. m, meters; s, s

This study takes advantage of the use of adeno-associated viral vector to overexpress Aha1, FKBP52 or mCherry (control) in an aged wild-type mouse. AAV serotype 9, used in this study, has been reported to target expression mainly in neurons and astrocytes with high transduction efficiency [26, 31]. Using AAV9 also overcomes some of the limitations offered by transgenic mice (genetic drift and disruptions) [52]. To determine if chaperone imbalance could initiate tau pathogenesis in the aged brain, we bilaterally injected 9-month-old wild-type mice with AAV9 to express mCherry, Aha1, or FKBP52 (N = 6 for each group) in the hippocampus (Fig. 1a). The use of AAV9 provided several advantages including a long-term stable expression of these chaperones without causing a chronic immune response in the brain at specifically selected timepoints [83]. In these mice, the virus was expressed for 7 months before exposing them to behavioral testing and, subsequently, collecting brain tissue at 16 months of age. Confirmation of viral overexpression by tissue quantification of mCherry, Aha1, and FKBP52 in the hippocampus is shown in Fig. 1b. Since neuropsychiatric symptoms like anxiety are common in early stage of AD [28], we first investigated whether our treatments increased anxiety-like behavior, while also assessing general motor ability to rule out confounding factors in our behavioral testing [88]. Overexpression of Aha1 or FKBP52 did not significantly affect mobility (measured by total distance) (Fig. 2a) or anxiety levels (measured by total time spent in the center) (Fig. 2b), although there was a trend toward an increase in anxiety-like behavior in Aha1 expressing mice when compared to control (#*p* = 0.07). Because the accumulation of tau is critically linked with the progression of dementia in AD patients [4, 13, 32, 70, 72, 73, 77, 79, 89], we assessed whether high levels of Aha1 or FKBP52 affect associative fear and spatial learning in aged wild-type mice. In the fear conditioning test, Aha1 injected mice spent significantly less time freezing during the last minute of the training day as compared to mCherry mice (Fig. 2c), suggesting that associative learning was impacted, but this did not result in contextual or cued memory deficits in the subsequent days. In the radial-arm water maze (RAWM) task no differences in learning and memory were identified during training (day 1) and testing (day 2) (Fig. 2d). On day 3, when increasing difficulty and testing for flexibility in learning a new spatial goal (reversal testing), FKBP52 injected animals made significantly more errors than control injected mice in locating the escape platform, indicating less flexibility and an impairment in reversal spatial learning. These results suggest that disrupted levels of FKBP52 and Aha1 alter cognitive ability of aged wild-type mice.

### High levels of Aha1 or FKBP52 increase unique tau phosphosites without affecting oligomeric tau in wild-type mice

**Fig. 3:**
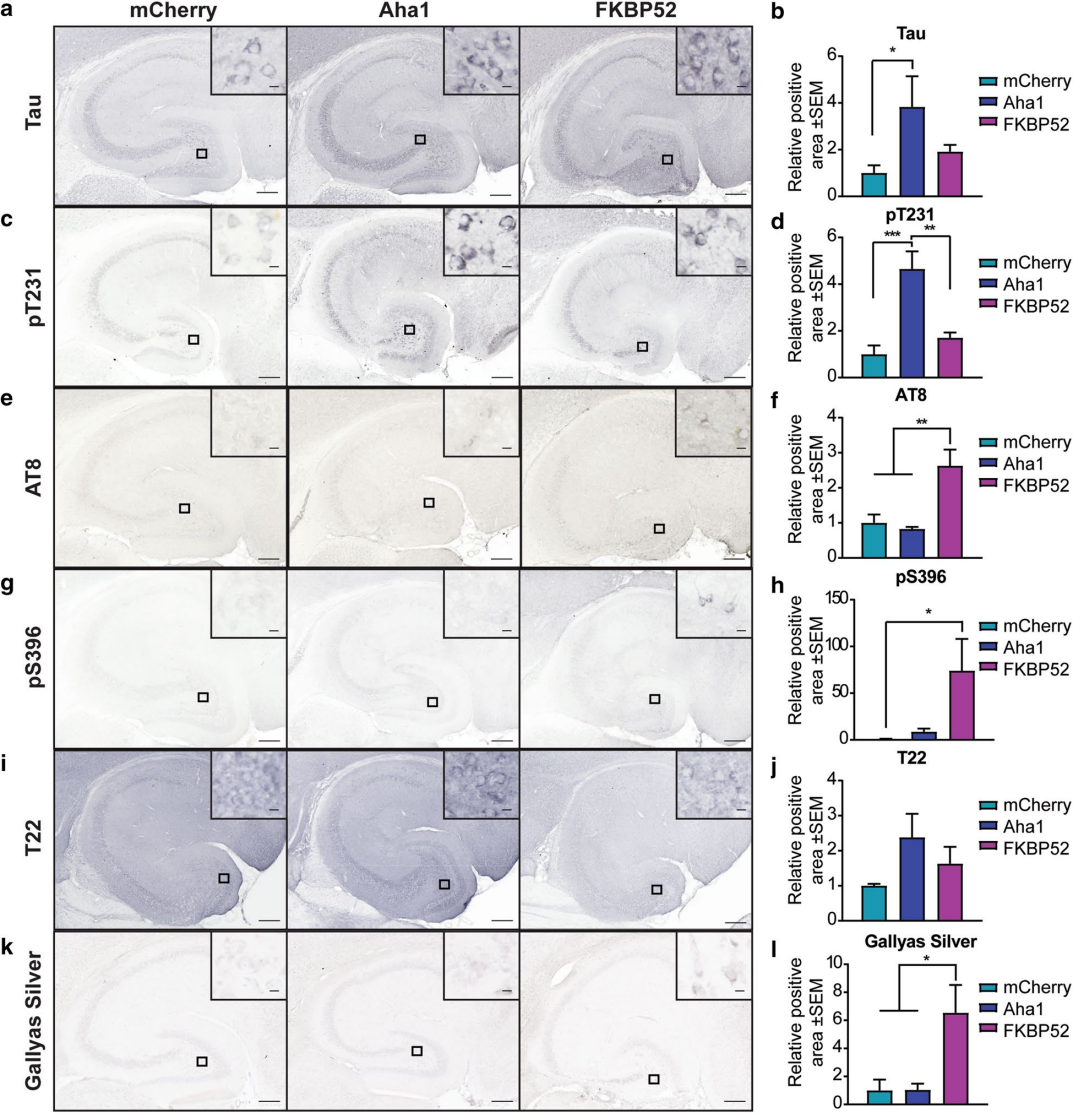
Overexpression of Aha1 or FKBP52 increases discrete phospho-tau species in aged wild-type mice. Representative images and analysis of hippocampal tissues from 16-months old wild-type mice expressing AAV9-mCherry, AAV9-Aha1, or AAV9-FKBP52 stained for (**a, b**) total tau (Dako) and phosphorylated tau species: (**c, d**) pT231 tau, (**e, f**) AT8 tau (pS202/T205), and (**g, h**) pS396 tau, as well as (**i, j**) T22 oligomeric tau and (**k, l**) Gallyas silver-positive tau. N = 6/AAV. Relative intensity was analyzed by one-way ANOVA followed by Tukey post-hoc test. Statistical significance is indicated by *p < 0.05, **p < 0.01 and ***p < 0.001. Results represented as standard error of the mean (± SEM). Scale bar represents 100 µm; inset scale represents 10 µm

Since we know these chaperones promote the aggregation of human tau [19, 90] and strong correlations between cognitive impairments and tau pathology have been reported in multiple studies [7, 72, 78, 87], we next determined if these chaperones promoted the accumulation of murine tau. We measured levels of total tau as well as phospho-tau species in the hippocampus of these mice. Total tau was significantly increased by Aha1 (Fig. 3a, b) as well the levels of pT231 tau (Fig. 3c, d). Conversely, high levels of FKBP52 promoted AT8 (pS202/T205) (Fig. 3e, f) and pS396 (Fig. 3g, h) tau phospho-species compared to mCherry injected mice. Oligomeric tau (T22) was not changed by high levels of these chaperones (Fig. 3i, j). Although no true tau tangles were observed, diffuse argyrophilic signal was significantly increased in the FKBP52 injected mice (Fig. 3k, l). Additional insets of CA1, CA3 and dentate gyrus representing these tissue analyses can be found in Additional file 1: Fig. S1 a–f.

**Fig. 4:**
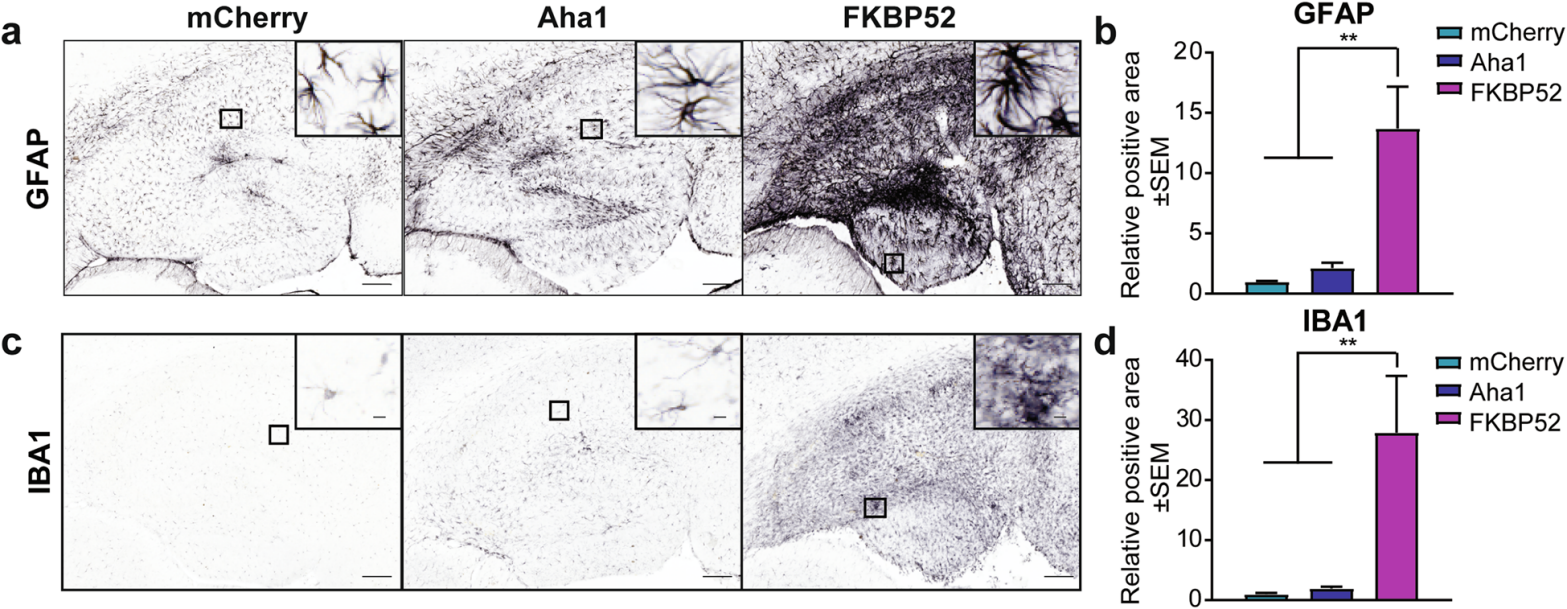
Examples of tau accumulation in the hippocampus of aged wild-type mice following overexpression of Aha1 or FKBP52. High magnification images (100x) were obtained from the hippocampus of a representative animal in the group with the highest tau accumulation. Representative images of tau species and their respective groups are the following: (**a**) total tau (Dako; AAV9-Aha1), (**b**) pT231 tau (AAV9-Aha1), (**c**) AT8 tau (pS202/T205; AAV9-FKBP52), (**d**) pS396 tau (AAV9-FKBP52), (**e**) T22 (AAV9-Aha1), and (**f**) Gallyas-silver (AAV9-FKBP52). Scale bar represents 10 µm

To gain a better understanding of the pattern of tau accumulation and how this relates to pathology in the tauopathic brain, we captured high magnification images from the hippocampus of these mice specifically in the group that showed the most accumulation of tau (Fig. 3a–l). Tau depositions in AAV9-Aha1 neurons were highly concentrated in the perinuclear space as well as some axonal distribution (Fig. 4a). The neuronal population stained for pT231 (AAV9-Aha1 tissue) and AT8 (AAV9-FKBP52 tissue) tau also showed perinuclear and axonal pre-tangle accumulation but was more heterogeneous (Fig. 4b, c). Particularly, AT8-positive cells showed a mixture of diffused and granular immunoreactivity (Fig. 4c). The presence of these mixed features was also evident in the smaller population of pS396-positive neurons (AAV9-FKBP52 tissue; Fig. 4d). The minimal T22-reactivity that could be detected was found to be perinuclear in a randomly selected tissue section from an AAV9-Aha1 injected mouse (Fig. 4e). We also noted the presence of argyrophilic-like grains in the silver immunoreactive neurons in high FKBP52 expressing animals (Fig. 4f). Overall, identification of these key morphological features in the pre-tangle stage provide valuable information on how these chaperones may participate in the evolution of tangle formation.

### Overexpression of FKBP52, but not Aha1, promotes gliosis and neuronal loss in aged mice

**Fig. 5:**
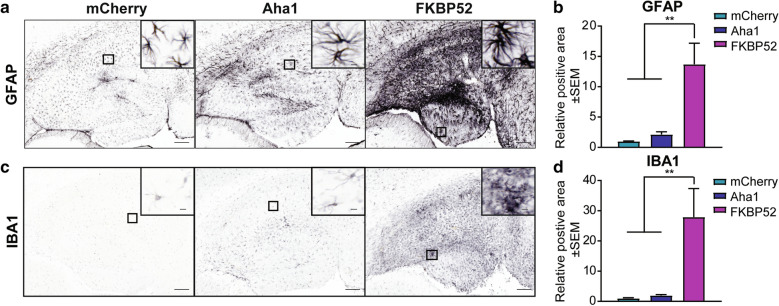
FKBP52 promotes activation of astrocytes and microglia in aged wild-type mice. Representative images and analysis of hippocampal tissues from 16-month-old wild-type mice expressing AAV9-mCherry, AAV9-Aha1, or AAV9-FKBP52 stained for (**a, b**) astrocyte marker (GFAP) and (**c, d**) microglial marker (IBA1). N = 6/AAV. Relative intensity was analyzed by one-way ANOVA followed by Tukey post-hoc test. Statistical significance is indicated by **p < 0.01. Results represented as standard error of the mean (± SEM). Scale bar represents 100 µm; inset scale represents 10 µm

In addition to tau accumulation, elevated markers of gliosis are commonly detected in the brains of aged individuals and further elevated in AD [9, 50, 54, 84]. Here, we investigated whether Aha1 or FKBP52 overexpression affected gliosis through the activation of the two main glial cells: astrocytes and microglia. Hippocampal tissues overexpressing mCherry, Aha1, or FKBP52 were stained for GFAP (glial filially acidic protein, astrocytic marker) and IBA1 (ionized calcium binding adaptor molecule 1, microglia marker). Glial cells remained relatively unaffected by Aha1 overexpression, however, FKBP52 significantly upregulated GFAP (Fig. 5a, b) and IBA1 (Fig. 5c, d) levels in the hippocampus, which implies a role for FKBP52 in promoting neuroinflammation in the aged brain, either dependently or independently on tau.

**Fig. 6:**
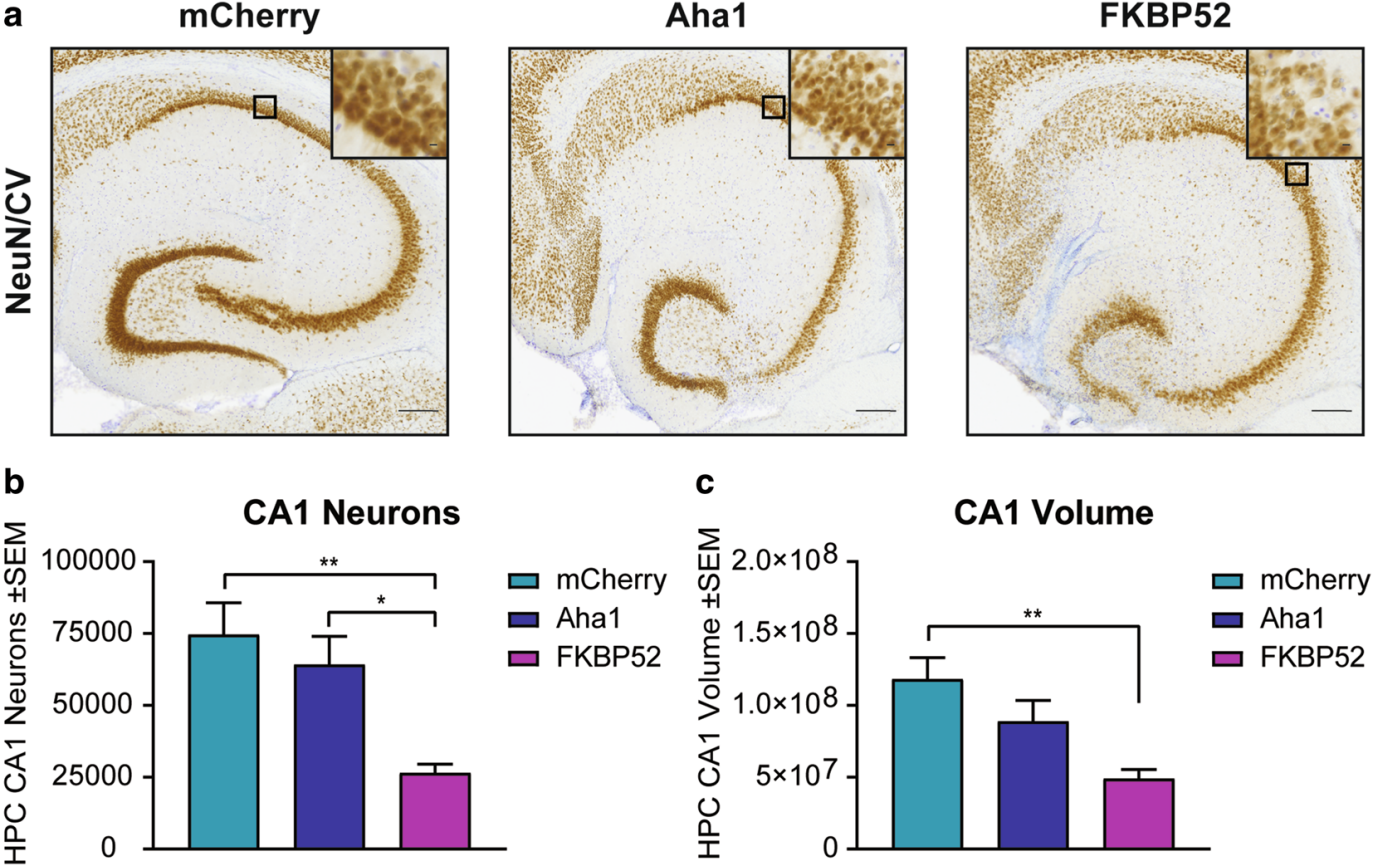
Overexpression of FKBP52 promotes neurotoxicity in aged wild-type mice. (**a**) Representative images from hippocampal neurons stained with NeuN (brown) and cresyl violet (purple). These hippocampal slices correspond to AAV9-mCherry, AAV9-Aha1, and AAV9-FKBP52 injected wild-type mice. Quantification of CA1 hippocampal (**b**) neuronal density and (**c**) volume using unbiased stereology from these animals. N = 6/AAV. Results represent the standard error of the mean (± SEM). Data was analyzed by a one-way ANOVA followed by Tukey post-hoc test. *p < 0.05 and **p < 0.01 is considered statistical difference in neuronal loss. Scale bars = 100 µm and inset scale represents 10 µm. CV, cresyl violet; CA1, Cornu ammonis subfield 1; HPC, hippocampus

**Fig. 7:**
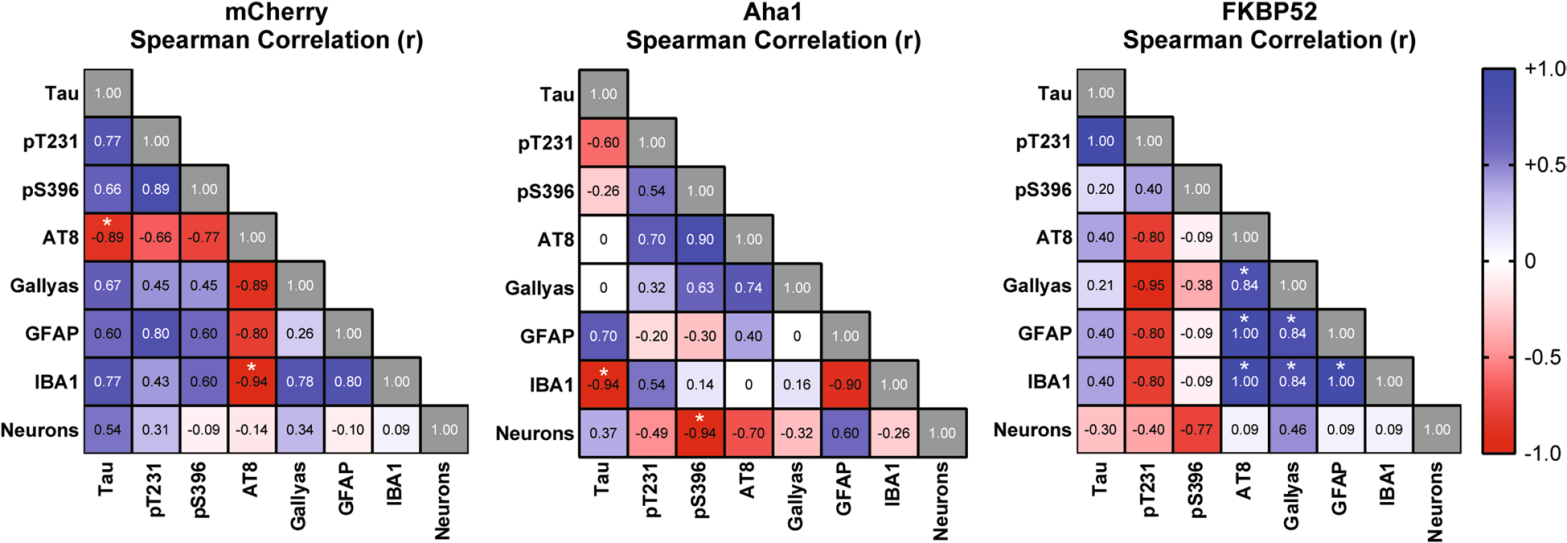
High levels of FKBP52 positively correlate with glia, AT8, and Gallyas silver positive tau. Spearman rank correlation matrix of proteins assessed in the hippocampus of aged wild-type mice are included in the analysis. N = 6/AAV. Red colors show strong negative correlations (− 1.0), blue shows strong positive correlations (+ 1.0), white shows no linear correlations (0), and grey represents correlation between the same readout. A value of *p < 0.05 indicates statistical significance

The exaggerated activation of glial cells as well as the cognitive deficits driven by FKBP52 denoted a possible detrimental effect on hippocampal neuron health. Using unbiased stereology, we examined the NeuN/cresyl violet-positive neuronal population in the hippocampal CA1 region (Fig. 6a). A significant neuronal loss (Fig. 6b) and reduced hippocampal volume (Fig. 6c) was observed in the FKBP52 injected animals. Neuronal number and tissue volume was not affected by Aha1 overexpression. Using these data, we performed a correlation analysis to understand the association between tau accumulation, glial activation, and neuronal density. T22 was excluded in this assessment because its expression was not affected by Aha1 or FKBP52 (Fig. 3). In general, the nonparametric Spearman rank correlation analysis shows a highly significant association of neuroglial cells with high levels of FKBP52 (Fig. 7). There were strong positive correlations between AT8 phospho-tau and Gallyas silver staining with FKBP52. This was different from mCherry and Aha1 animals where only weak associations were found with IBA1 and AT8 and pS396 phospho-tau species. These data indicate that FKBP52 may be a potential driver of unique pathological events, like AT8 tau accumulation and activation of neuroglial cells, which may evoke a negative downstream effect on neuronal health in the hippocampus.

### FKBP52 triggers the accumulation of AT8 tau and markers of neurogliosis in hippocampal adjacent cortices

**Fig. 8:**
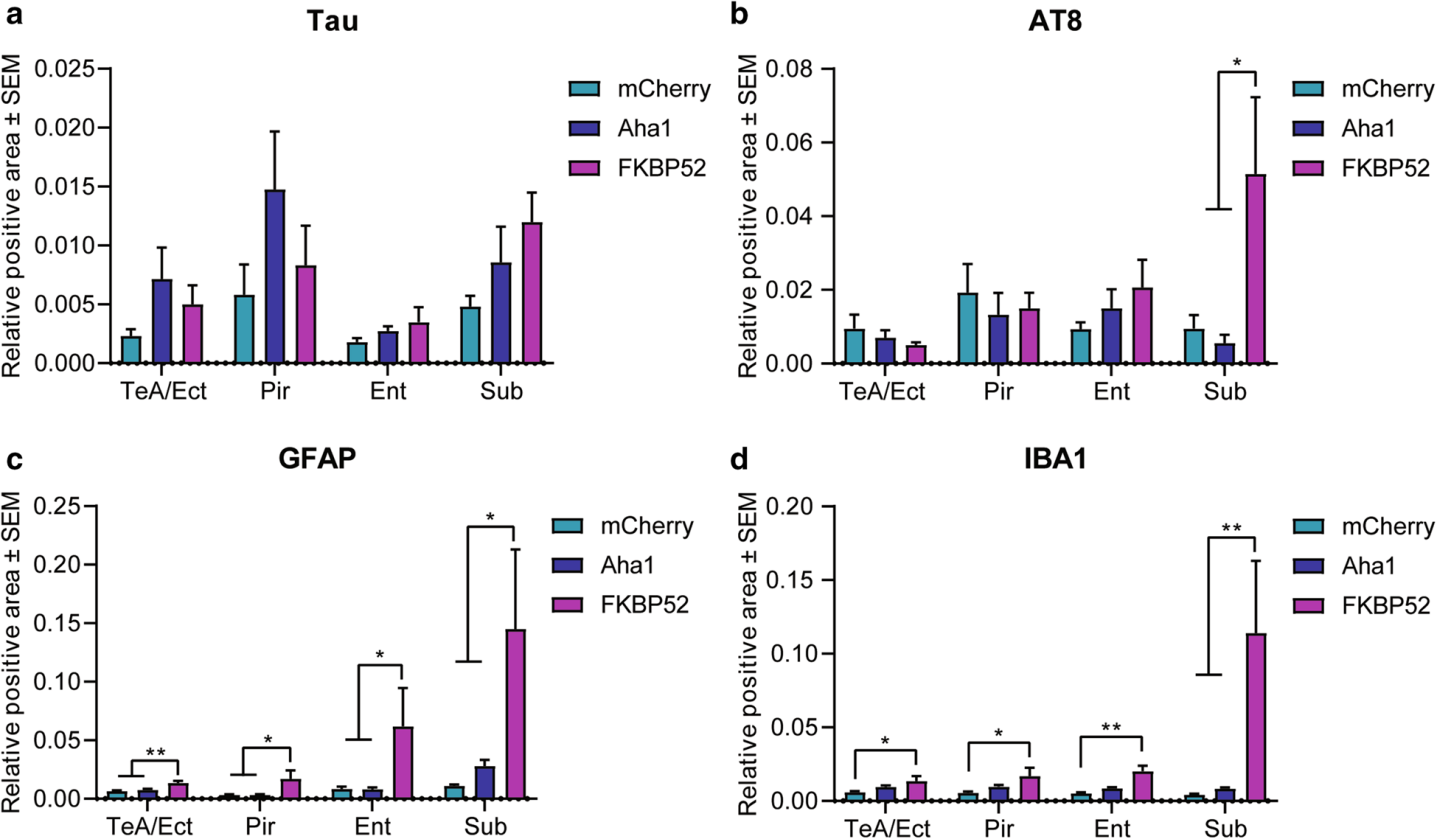
FKBP52 induces gliosis in hippocampal adjacent cortices without affecting total tau expression. Analysis of cortical subregions from 16-month-old wild-type mice expressing AAV9-mCherry, AAV9-Aha1, or AAV9-FKBP52 stained for (**a**) tau, (**b**) AT8 (pS202/T205), (**c**) astrocyte marker (GFAP), and (**d**) microglial marker (IBA1). N = 6/AAV. Relative intensity was analyzed by one-way ANOVA followed by Tukey post-hoc test for each subregion. Statistical significance is indicated by *p < 0.05 and **p < 0.01. Results represented as standard error of the mean (± SEM). TeA/Ect, temporal association cortex and ectorhinal cortex; Pir, perirhinal cortex; Ent, entorhinal cortex; Sub, subiculum

In addition to the hippocampus, cortical areas play a key role in cognition and are vulnerable to tau pathology and neurodegeneration [24]. Here, we examined the presence of astrocyte and microglial markers in areas adjacent to the hippocampus. Aha1 or FKBP52 did not affect total tau levels in the ectorhinal cortex, perirhinal cortex, entorhinal cortex or the subiculum (Fig. 8a). Because FKBP52 induced AT8 tau in the hippocampus, we also examined its expression in these cortices. FKBP52 increased the levels of this phospho-tau species in the subiculum (Fig. 8a), which was also the area showing the highest induction in markers of GFAP (Fig. 8b) and IBA1 (Fig. 8c) driven by FKBP52. Data indicate that FKBP52 contributes not only to the induction of these glial cells and tau accumulation but also the spreading of these pathologies in the brain.

## Discussion

The impact of high levels of Aha1 or FKBP52 in the aged brain was examined in this study. We showed that high expression of either chaperone increases pathological tau species in 16-month-old wild-type mice. We also found that Aha1 overexpression impaired associative learning while FKBP52 overexpression caused deficits in cognitive flexibility during spatial learning. In addition, high FKBP52 levels evoked a significant neuroinflammatory response of both GFAP and IBA1 not only in the hippocampus, but in cortical areas adjacent to the injection site where tau was also found to accumulate. We found a strong correlation between levels of FKBP52, glial cell markers, and AT8 tau, which may be key players driving the neuronal loss and cognitive deficits observed in these mice.

Most preclinical studies have focused on recapitulating tau pathology in vitro or by using transgenic mice [39, 40, 60]. Although these strategies have been very valuable for understanding biological mechanisms underlying the disease, their translatability in clinical trials has been questioned [25, 39]. Our study excluded the most common limitations and confounding effects of using tau transgenic mouse models, which include the insertion of transgenes, the deletion of endogenous genes, the overexpression of tau, the inclusion of tau mutations, and the limited ability to evaluate age-dependent neurodegeneration. Another advantage presented in this study is the ability to maintain a long-term stable expression of these chaperones in neurons starting at specific timepoints by AAV9 transduction. This initiated a pathogenic cascade that involved tau accumulation instead of tau being the initiating event, which may better represent the tauopathic brain in diseases, like AD, which are absent of tau mutations and total tau levels are unchanged. Our results are highly relevant to age-related effects in normal and pathological aging, since tau pathology can occur in the brains of individuals from both groups [30]. Prior studies from our group revealed that Aha1 and FKBP52 chaperones also promote the accumulation of tau in rTg4510 tau transgenic mice [19, 90]. However, in these studies, no significant effects of Aha1 or FKBP52 overexpression on cognition or pathology were observed in young wild-type mice. This really highlights the importance of the aging component, since short-term (2–3 month) overexpression of these molecular chaperones in younger (5–6 month) animals did not initiate pathogenesis as in our current study.

Our findings suggest that chaperone imbalance could initiate and promote neurodegeneration in aged wild-type mice via two pathways: by inducing tau accumulation and by promoting a neuroinflammatory state in the hippocampus. These processes are interconnected; hence they can evoke a feedforward loop once activated [61]. Both Aha1 and FKBP52 increased phosphorylation of discrete tau species. However, only FKBP52 overexpression caused neurodegeneration, suggesting that FKBP52 initiates a more aggressive pathological cascade. FKBP52 overexpression increased AT8 and pS396 phospho-tau species as well as argyrophilic-like tau-positive signal. Based on our examination, FKBP52 may participate in the pre-tangle stage by inducing structural features often described in stages preceding neurofibrillary tangle formation when neuronal loss is occurring [60, 61, 98]. Some of our observations include the presence of the perinuclear accumulation of total and phospho-tau species, diffuse and granular tau immunoreactivity, and argyrophilic-like grains. However, no tau tangles were observed. We may not see tangles in these brains because of the biological differences between human and murine tau. The normal evolution from pre-tangles to mature neurofibrillary tangles requires a shift between two tau isoforms: the 3R and 4R [99]. Different from humans, adult mouse brain lacks 3R tau, which is thought to be needed for maturity of fibrils and tangles in AD [47, 99].

Since FKBP52 levels were reported as low at advanced pathological stages [38], based on what this study revealed perhaps this is caused by the loss of neurons expressing high levels FKBP52 earlier in the disease. A heighten inflammatory response is likely also contributing to the neurotoxicity in the FKBP52 injected wild-type mice. This stimulation could be through FKBP52- or tau-mediated mechanisms. FKBP52 is known to promote a proinflammatory environment through the activation of the NF-κB and the IL-6 cytokine [29]. Increased levels of these proteins may be responsible for inducing GFAP and IBA1 [94], correlating with our observations in mice receiving FKBP52 injections. FKBP52 also potentiated activation of glial cells in areas adjacent to the hippocampus escalating the hippocampal-cortical damage. These observations indicate that FKBP52 may be responsible for maintaining a continuous inflammatory state by promoting proinflammatory markers, which may lead to the release of neurotoxic factors from astrocytes and microglia [64], ultimately causing neuronal damage.

Because FKBP52 and Aha1 are both co-chaperones of Hsp90, disequilibrium or disruption of existing Hsp90 chaperone heterocomplexes may be another factor contributing to the pathogenesis in these mice. FKBP52 competes for binding the tetratricopeptide repeat (TPR) domain of Hsp90 with other co-chaperones including FKBP51, Cyp40, and CHIP [8, 75]. The effect of FKBP52 on tau accumulation may be exacerbated by the displacement of these other chaperones, since some co-chaperones, like Cyp40 and CHIP, have been shown to prevent tau accumulation [5, 23]. Although Aha1 binds to a distinct region of Hsp90, Aha1 has been shown to regulate the binding of other co-chaperones to Hsp90 by preventing the binding of HOP, p50, and Cdc37 [41, 96]. Hsp90 heterocomplexes are key regulators of steroid hormone receptors. The GR complex, for example, is regulated by FKBP52 levels. FKBP52 promotes its nuclear translocation while competing with FKBP51, which slows GR transactivation [97]. This will not only generate a greater GR-driven response to stress, but also promotes transcription of pro-inflammatory cytokines [7]. Binding of HOP, Hsp70, and p23 clients to Hsp90-FKBP52-GR complex can also determine the steroid receptor maturation and activity affecting stress response as well as the chaperone interaction with tau [48, 85]. Interestingly, Aha1 competes with GR for Hsp90 binding site, ultimately inhibiting GR activity [66, 80]. This may explain the lower inflammatory reaction in Aha1 injected animals, despite the increase in pT231 tau. Further studies must examine how these chaperone heterocomplexes are affected and if there is a feedforward potentiation among specific chaperones, tau, and glial activation.

Declined proteostasis in aging limits the chaperone capacity to remodel proteins to their native form or direct them towards degradation [56]. We know that, in the hyperphosphorylated state, tau undergoes polyubiquitination, but evades degradation by the proteasome [51]. Thus, it is possible that FKBP52-induced levels of phospho-tau species intensified the burden on the aged proteostasis system, causing the neurotoxicity and neuronal loss seen in this study. This is in line with a previous study implicating a role for FKBP52 in neuronal health where overexpression of FKBP52, not only destabilizes microtubules by affecting tau, but also reduces neurite outgrowth in PC12 cells [17, 18]. The effects we measured on tau and neuronal loss could also be an indication of failure in other degradation systems, like the autophagy system [57], since others have reported tau accumulation, neuroinflammation and neuronal loss in mice lacking the autophagy protein, Atg16L1 [46].

Future research is needed to address the current limitations of this study. First, the limited number of aged animals that were available to use for this study makes it difficult to evaluate sex differences due the small representation of sex in each group. Increasing the sample size will provide further confidence in the behavioral and pathological effects described here as well as allow for the examination of sex-difference. Second, it is difficult to determine the order of events by which FKBP52 causes neuronal loss. By assessing earlier timepoints after AAV9 injections in aged wild-type as well as in tau knockout mice, the timing and relationship between tau phosphorylation, inflammation, and neuronal loss could be examined. Third, we were expecting an accumulation of tau oligomers by Aha1 and FKBP52, based on our prior studies in tau transgenic mice [19, 90]. Failure to detect these oligomers may be due methodological limitations since the use of these antibodies on mouse tau has not been characterized. Further testing of specific antibodies against mouse tau oligomers is needed to confirm the null effect on oligomers. Fourth, examining how other Hsp90 heterocomplexes may be affected by high levels of FKBP52 or Aha1 would provide important information about how declined proteostasis is sustained during aging. Fifth, testing additional molecular chaperones may reveal members that drive unique pathologies as well as discrete chaperones absent of any effect, which could be used as controls in future studies. Lastly, we observed a significant silver-positive signal in FKBP52 injected mice without the formation of true tau tangles. Additional studies are needed to determine if FKBP52 overexpression could drive tangles at later timepoints in aged wild-type mice or if the lack of 3R tau in the aged mouse brain prevents mature tangle formation [47]. It is possible that this pathology may only be generated by FKBP52 in mice expressing human tau.

In conclusion, our findings demonstrate that imbalances in molecular chaperones can have a significant impact on the pathogenic accumulation of endogenous tau in the aged mouse brain. Both Aha1 and FKBP52 altered tau accumulation and cognition, but only FKBP52 induced gliosis in the hippocampus and adjacent brain cortices. Our discoveries suggest that FKBP52 may aggressively promote changes in tau, activate glial cells, and this in turn may cause a neurotoxic environment for neurons. The initiation of these pathologies in one brain region caused aberrant accumulation in connected brain regions, which may model the prion-like spreading of tau that has been described in AD. This demonstrates that disturbances in molecular chaperone proteins in an aged wild-type mouse brain models key features of AD-like pathogenesis.

## Supplementary Information

**Additional file 1. Fig. S1:** Overexpression of Aha1 or FKBP52 increases discrete phospho-tau species in aged wild-type mice. Additional insets from Figure 3 showing the CA1, CA3, and dentate gyrus (DG) from 16-months old wild-type mice expressing AAV9-mCherry, AAV9-Aha1, or AAV9-FKBP52 stained for (a) total tau (Dako), (b) pT231 tau, (c) AT8 (pS202/T205) tau, and (d) pS396 tau, as well as (e) T22 oligomeric tau and (f) Gallyas silver-positive tau. Scale bar represents 10 μm

## Data Availability

The datasets used and/or analyzed during the current study are available from the corresponding author on reasonable request.
