# Correction to: Hsp90 co‑chaperones, FKBP52 and Aha1, promote tau pathogenesis in aged wild‑type mice

**DOI:** 10.1186/s40478-021-01188-5

**Published:** 2021-05-11

**Authors:** Marangelie Criado-Marrero, Niat T. Gebru, Danielle M. Blazier, Lauren A. Gould, Jeremy D. Baker, David Beaulieu-Abdelahad, Laura J. Blair

**Affiliations:** 1 USF Health Byrd Alzheimer’s Institute, University of South Florida, Tampa, FL 33613 USA; 2 Department of Molecular Medicine, Morsani College of Medicine, University of South Florida, Tampa, FL 33620 USA; 3 Research Service, James A Haley Veterans Hospital, 13000 Bruce B Downs Blvd, Tampa, FL 33612 USA

## Correction to: Criado-Marrero et al. acta neuropathol commun (2021) 9, 65 https://doi.org/10.1186/s40478-021-01159-w

After publication of this article [1], it is noticed this article contained an error: Figure 4 was incorrect.

The correct Fig. 4 has been provided in this Correction.

**Fig. 4:**
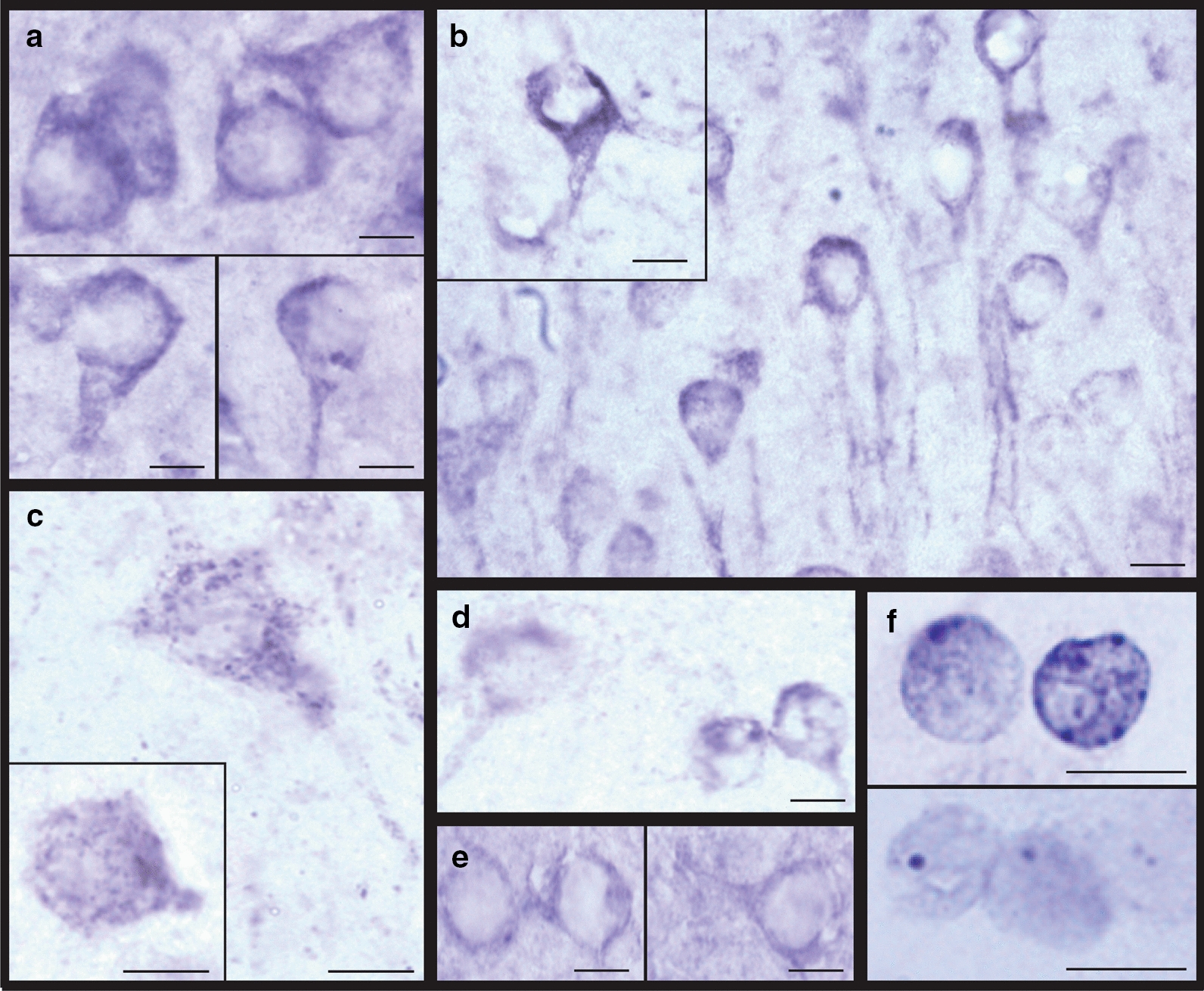
Examples of tau accumulation in the hippocampus of aged wild‑type mice following overexpression of Aha1 or FKBP52. High magnification images (×100) were obtained from the hippocampus of a representative animal in the group with the highest tau accumulation. Representative images of tau species and their respective groups are the following: (**a**) total tau (Dako; AAV9‑Aha1), (**b**) pT231 tau (AAV9‑Aha1), (**c**) AT8 tau (pS202/T205; AAV9‑FKBP52), (**d**) pS396 tau (AAV9‑FKBP52), (**e**) T22 (AAV9‑Aha1), and (**f**) Gallyas‑silver (AAV9‑FKBP52). Scale bar represents 10 µm

The original article has been updated.
