# Supplementary material for: Hsp90 co-chaperones, FKBP52 and Aha1, promote tau pathogenesis in aged wild-type mice
Source: Acta Neuropathol Commun. 2021 Apr 8;9:65. doi: 10.1186/s40478-021-01159-w (PMC8033733; doi:10.1186/s40478-021-01159-w)
Supplement: Supplementary file 1 — Additional file 1. Fig. S1: Overexpression of Aha1 or FKBP52 increases discrete phospho-tau species in aged wild-type mice. Additional insets from Figure 3 showing the CA1, CA3, and dentate gyrus (DG) from 16-months old wild-type mice expressing AAV9-mCherry, AAV9-Aha1, or AAV9-FKBP52 stained for (a) total tau (Dako), (b) pT231 tau, (c) AT8 (pS202/T205) tau, and (d) pS396 tau, as well as (e) T22 oligomeric tau and (f) Gallyas silver-positive tau. Scale bar represents 10 μm [file 40478_2021_1159_MOESM1_ESM.docx]

**Hsp90 co-chaperones, FKBP52 and Aha1, promote tau pathogenesis in aged wild-type mice**

**Marangelie Criado-Marrero**^1,2^**, Niat T. Gebru**^1,2^**, Danielle M. Blazier**^1,2^**, Lauren A. Gould**^1,2^**, Jeremy D. Baker**^1,2^**, David Beaulieu-Abdelahad**^1,2^**, Laura J. Blair**^1,2,3^

^1^USF Health Byrd Alzheimer’s Institute, University of South Florida, Tampa, FL 33613, USA

^2^Department of Molecular Medicine, Morsani College of Medicine, University of South Florida, Tampa, FL 33620, USA

^3^Research Service, James A Haley Veterans Hospital, 13000 Bruce B Downs Blvd, Tampa, FL 33612, USA

Marangelie Criado-Marrero and Niat T. Gebru have contributed equally to this work.

Corresponding author

Laura J. Blair, PhD

(813) 396-0639

[laurablair@usf.edu](mailto:laurablair@usf.edu)


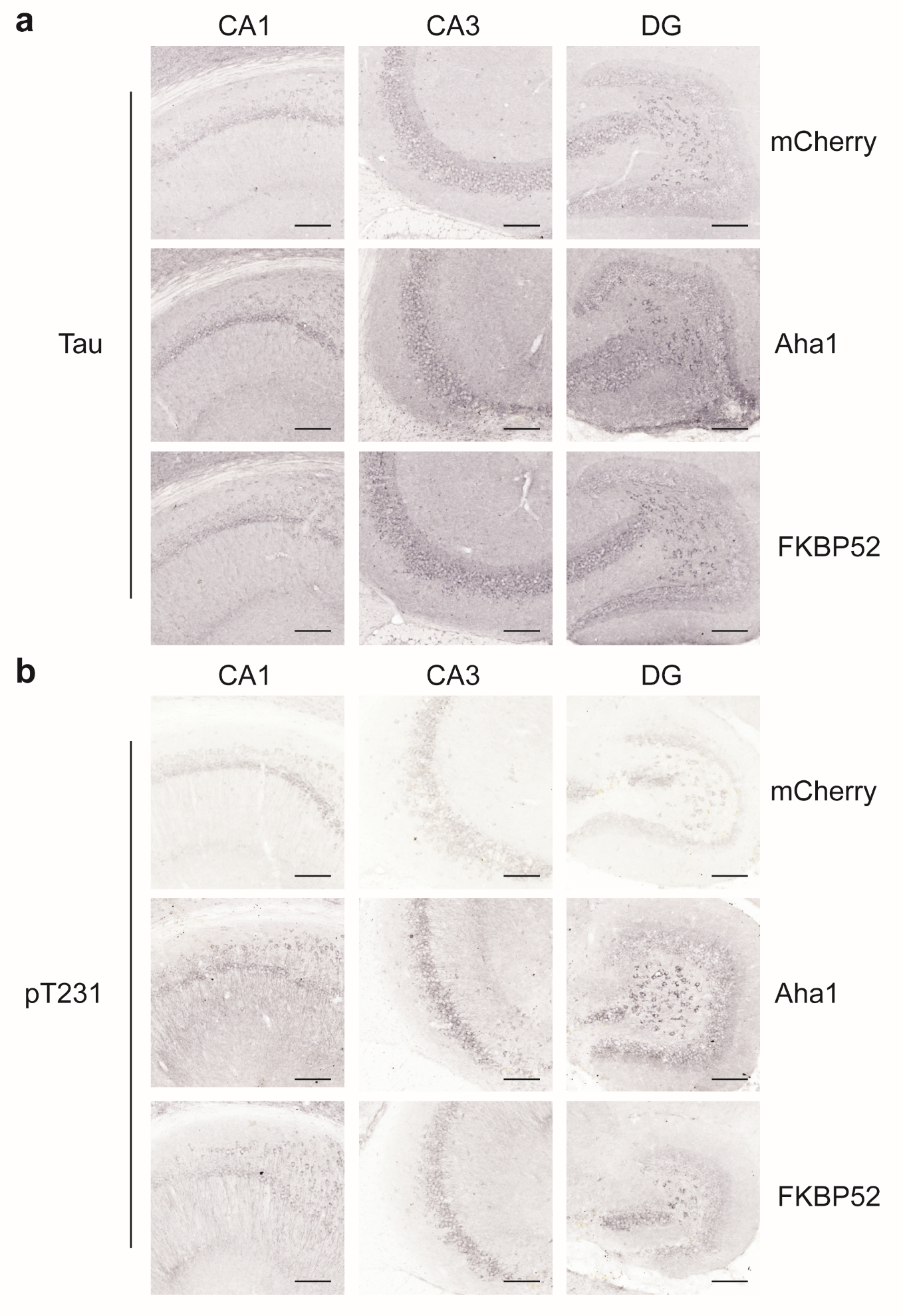


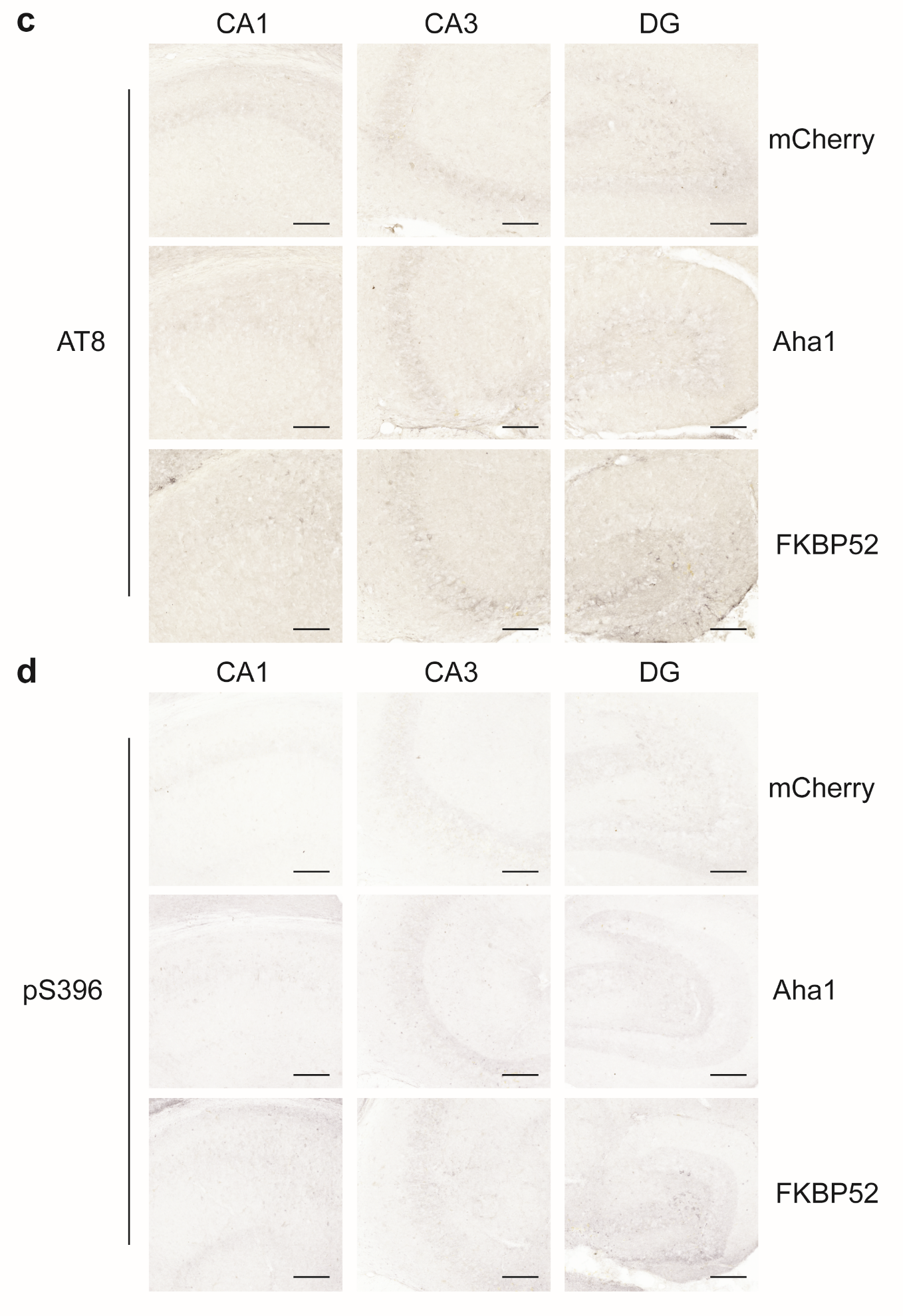


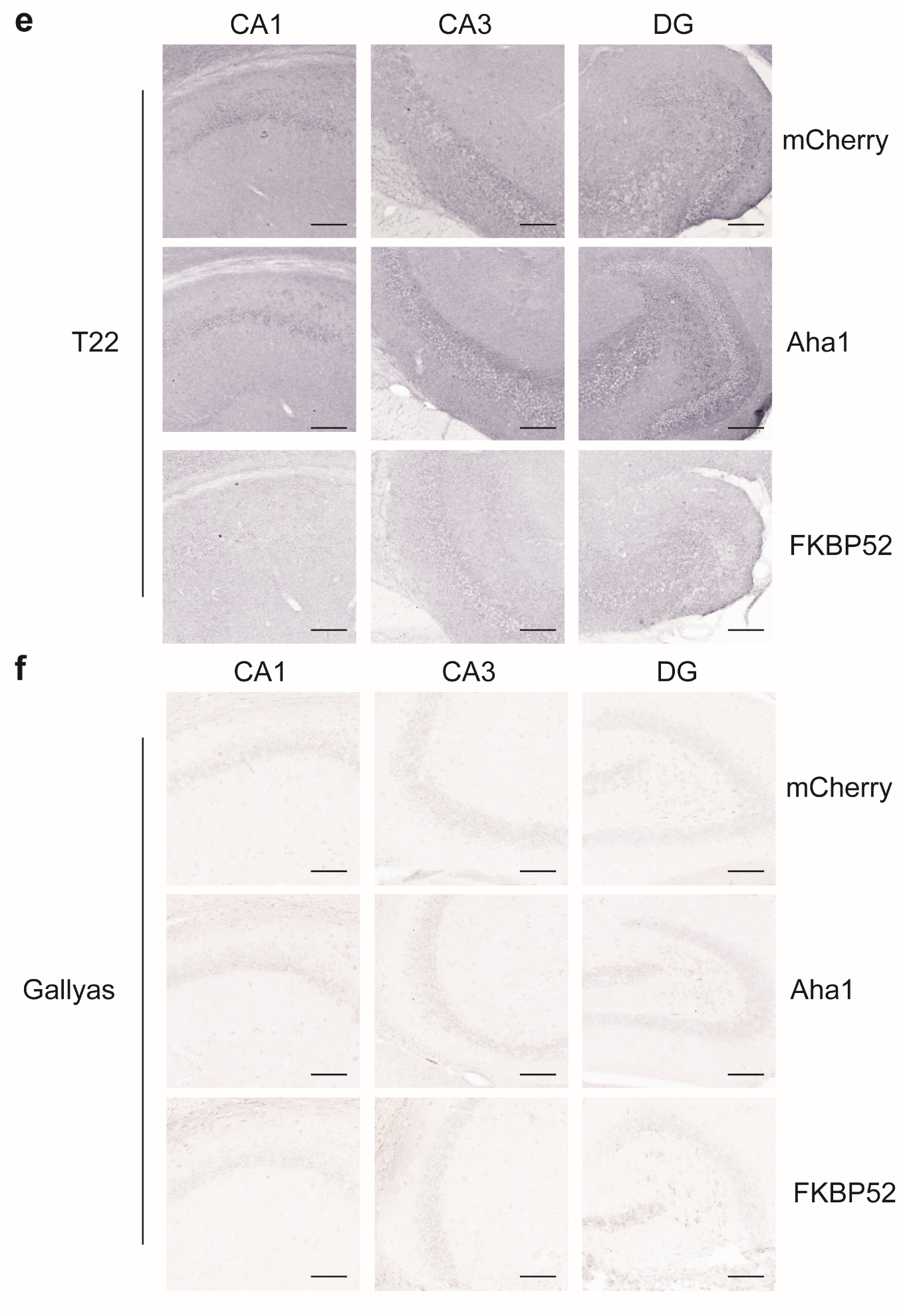


**Fig. S1 Overexpression of Aha1 or FKBP52 increases discrete phospho-tau species in aged wild-type mice.** Additional insets from Figure 3 showing the CA1, CA3, and dentate gyrus (DG) from 16-months old wild-type mice expressing AAV9-mCherry, AAV9-Aha1, or AAV9-FKBP52 stained for (**a**) total tau (Dako), (**b**) pT231 tau, (**c**) AT8 (pS202/T205) tau, and (**d**) pS396 tau, as well as (**e**) T22 oligomeric tau and (**f**) Gallyas silver-positive tau. Scale bar represents 10 µm.
